# Investigation on sleep quality and psychological distress in patients with pulmonary nodules

**DOI:** 10.1186/s40359-023-01274-4

**Published:** 2023-09-01

**Authors:** Junhan Wu, Weitao Zhuang, Haijie Xu, Yong Tang, Shaopeng Li, Wei Xu, Xuefeng Sun, Xianglin Li, Guibin Qiao

**Affiliations:** 1 Shantou University Medical College, Shantou, 515041 China; 2 Department of Thoracic Surgery, Guangdong Provincial People’s Hospital (Guangdong Academy of Medical Sciences), Southern Medical University, Guangzhou, China; 3 Department of Medical Oncology, State Key Laboratory of Oncology in South China, Collaborative Innovation Center for Cancer Medicine, Sun Yat-Sen University Cancer Center, Guangzhou, China; 4 Department of Thoracic Surgery, The First Affiliated Hospital of Shantou University Medical College, Shantou, China; 5 The Second School of Clinical Medicine, Southern Medical University, Guangzhou, China; 6 School of Public Health, Chongqing Medical University, Chongqing, China

**Keywords:** Anxiety, Depression, Pulmonary nodules, Sleep quality

## Abstract

**Background:**

Patients with pulmonary nodules (PNs) often suffer from the psychological burden of their disease and trap in sleep problems. This is insufficiently identified and addressed in clinical practice. The aim of this study was to investigate the psychological distress and sleep quality among PN patients and identify potential risk or protective factors for sleep quality.

**Methods:**

We conducted a cross-sectional study, which included 731 PN patients who visited the thoracic clinic of Guangdong Provincial People’s Hospital. Each participant completed a structured questionnaire consisting of demographic characteristics, clinical characteristics, the Hospital Anxiety and Depression Scale (HADS) and the Pittsburgh Sleep Quality Index (PSQI). The reliability of the HADS (Cronbach’s α = 0.944) and PSQI (Cronbach’s α = 0. 0.757) in this study was satisfactory.

**Results:**

A total of 328 patients (44.9%) had PSQI global scores > 5, indicating poor quality of sleep. Age ≥ 50 years (OR 1.88, 95% CI 1.35–2.58; *P* < 0.001), female (OR 1.56, 95% CI 1.05–2.33; *P* = 0.028), detection of nodule for 7–12 months (vs for more than 24 months, OR 2.14, 95%CI 1.18–3.89, *P* = 0.013), anxiety (OR 1.78, 95% CI 1.17–2.71; *P* = 0.007) and depression (OR 1.84, 95% CI 1.16–2.92; *P* = 0.010) were independent risk factors for impaired sleep quality. A significant correlation revealed that sleep quality was positively correlated with both anxiety and depression (Spearman r = 0.342, *P* < 0.001 and Spearman r = 0.314, *P* < 0.001, respectively). All dimensions of the PSQI scale were significantly decreased in both anxiety group and depression group compared to the psychologically normal group (*P* < 0.05).

**Conclusions:**

Impaired sleep quality is highly prevalent among patients with PNs and associated with age, gender, time from the date of detection, anxiety and depression. Based on the finding of impaired sleep quality and psychological health, screening for psychological and sleep problems in PN patients will be of great clinical benefit.

## Background

With the widespread application of low-dose computer tomography (LDCT) lung cancer screening, the detection rate of pulmonary nodules (PNs) has rapidly increased [1]. Although most of the PNs are benign in nature, misconception is commonly seen in individual patients, which might lead to overestimation of cancer risk [2] and result in nodule-related distress including anxiety and depression [3, 4]. For quite a few patients, this distress was severe enough to significantly impair their quality of life, such as daily activities and sleep quality, etc. [5].

Good sleep quality has been reported to be one of the important factors for maintaining immunity, including antitumor immune response [6]. For lung adenocarcinoma manifesting as a subsolid nodule, recent evidence had revealed an equilibrium phase of immunoediting in tumor microenvironment [7], which may explain the indolent behavior of these nodules. Nonetheless, chronic sleep disturbance may break this equilibrium, impair the antitumor immune response and stimulate the growth of the dormant nodules [8]. In this regard, the necessity to investigate the sleep disturbance and the associated psychological distress among the PN population cannot be over-emphasized, yet no study has been conducted to date to address this vital concern. Of note, assessment of sleep quality and psychological states has gain considerable attention in other nodule-related disease. Recently, Ruicen Li et al. [9] reported psychological distress and sleep disturbance were observed in patients with suspicious or malignant thyroid nodule using well-validated self-reported questionnaires. Currently, although much work had been done to investigate the psychological distress in patients with PNs, the condition of sleep quality and the interaction between psychological distress and sleep quality in these groups of PN patients need further exploration. In this study, we adopted the Patient Reported Outcome Measures (PROMs) for outcome assessment, which has been widely promoted to capture patients’ health status and to drive quality improvement in healthcare. The purposes of our study are to investigate the prevalence of psychological distress and impaired sleep quality among PN patients and to identify the psychological and clinical factors associated with impaired sleep quality. The results of the study will also provide evidence and hints for better patient-centered care and raise the attention to the psychological health among patients with PNs.

## Methods

### Participants

This study was a questionnaire-based cross-sectional study performed at the Thoracic Clinic of Guangdong Provincial People’s Hospital from Jan 1, 2021 to March 30, 2022. The inclusion criteria were as follows: (1) patients who were diagnosed with solitary or multiple ≤ 3 cm PNs using high-resolution CT scan, (2) aged eighteen years and over, (3) ability to complete the questionnaires and provide informed consent. The exclusion criteria included: (1) known pathological diagnosis of pulmonary nodules by biopsy or surgery, (2) previously diagnosed mental disorder, (3) previous history of malignancy on any site of the body.

### Questionnaires

The questionnaire consisted of three parts: demographic and clinical characteristics, psychological distress, and sleep quality. The participants were invited to complete the online questionnaires under the guidance of research assistants when they visited the online or offline outpatient clinic. Patients who did not have access to the internet completed their questionnaires using available tablets and paper or through oral survey.

Demographic features and clinical characteristics were collected, including age, gender, smoking status, family history of malignancy, subjective symptoms and time from the date of diagnosis. Two reviewers with consolidate experience reviewed all CT images and collected the imaging characteristics of nodules.

The Hospital Anxiety and Depression Scale (HADS) consists of two subscales used to assess anxiety and depression, and is a commonly used and reliable self‐reporting tool in clinical practice. Each subscale is composed of seven 4-point Likert scale questions, with the total score ranging from 0 to 21 and scores of 8 or greater indicating the presence of anxiety or depression [10–12], and showed a good internal consistency in this study (Cronbach’s α = 0.944).

Pittsburgh Sleep Quality Index (PSQI) [13] was used to assess sleep quality in participants. The PSQI comprises 19 self-rated questions grouped into 7 components (subjective sleep quality, sleep latency, sleep duration, habitual sleep efficiency, sleep disturbances, use of sleep medications, and daytime dysfunction). Each component score weighs equally on a 0–3 points scale. The scores of 7 components are summed up to a global PSQI score, which ranged from 0 to 21 points with a cutoff score of ≤ 5 indicating good sleep quality. This scale demonstrated acceptable reliability in this study (Cronbach’s α = 0.757).

### Statistical analysis

Demographic and clinical characteristics are described using mean ± standard deviation (SD) for continuous variables and frequencies (percentage) (*n*, %) for categorical variables. Shapiro–Wilk test was performed to check for the normality distribution of the data. In our study, the dependent variable is the sleep quality, which are divided into two grades. Therefore, the Mann–Whitney U test and the chi-square test were used to analyze the differences between different groups. Nonparametric tests were used for analysis of continuous variables among different groups whenever the data distribution did not meet the normality criteria. Variables with *P* ≤ 0.1 at univariable analysis were included in a multivariable logistic regression model. Moreover, correlation between continuous variables was calculated using the Spearman correlation test. All statistical analyses were performed using SPSS 25.0 (IBM SPSS Statistics for Windows. Armonk, NY: IBM Corp). Statistical significance was based on two-tailed *P*-values, with a value of *P* < 0.05 being statistically significant.

### Standard protocol approvals, registrations, and patient consents

This study was approved by the Research Ethics Committee of Guangdong Provincial People’s Hospital (KY-Q-2021–005-03) and was conducted in accordance with the Declaration of Helsinki (as revised in 2013). This study is part of the registered study in ClinicalTrial.gov (registration no. NCT04857333). Informed consent was obtained from all patients during the questionnaire distribution period.

## Results

### Prevalence of impaired sleep quality in PN patients

**Fig. 1:**
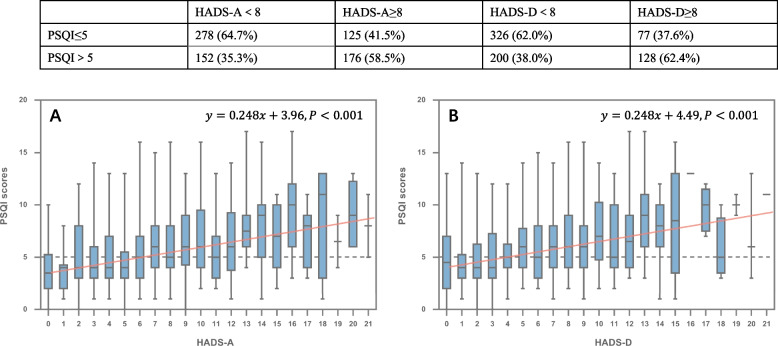
Associations between sleep quality and anxiety or depression burden in patients with pulmonary nodules. **A** HADS-A scores in respect to PSQI scores. **B** HADS-D scores in respect to PSQI scores. HADS-A, hospital anxiety and depression subscale-anxiety; HADS-D, hospital anxiety and depression subscale-depression; PSQI, Pittsburgh Sleep Quality Index

The mean PSQI score of 731 PN patients was 5.82 ± 3.27. Among them, 328 patients (44.9%) had a PSQI score > 5, which indicated impaired sleep quality. As shown in Fig. 1, 176 (58.5%) patients with HADS-A scores ≥ 8 reported impaired sleep quality, while 128 (62.4%) patients with HADS-D scores ≥ 8 reported impaired sleep quality. Conversely, significantly lower prevalence of impaired sleep quality was observed in patients with HADS-A scores < 8 (*n* = 152, 35.3%) or HADS-D scores < 8 (*n* = 200, 38.0%).

### Association between psychological distress and sleep quality

**Fig. 2:**
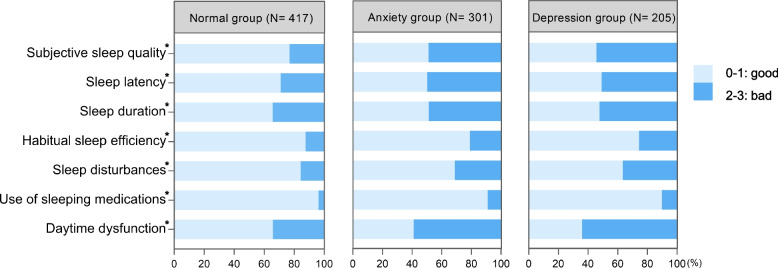
Comparison of different dimensions of PSQI between psychological normal group and anxiety or depression group in patients with pulmonary nodules. PSQI, Pittsburgh Sleep Quality Index; * *P* < 0.05

A correlation analysis revealed that sleep quality was significantly correlated with both anxiety and depression states (Spearman r = 0.342, *P* < 0.001 and Spearman *r* = 0.314, *P* < 0.001, respectively), with two fitted curves demonstrating similar trends (Fig. 1). It can be inferred that patients will probably suffer from impaired sleep quality when anxiety score reached six or higher and depression score reached four or higher. As shown in Fig. 2, the anxiety group and depression group presented significantly worse results in all the dimensions of the PSQI scale than the normal group (*P* < 0.05), including subjective sleep quality, sleep latency, sleep duration, habitual sleep efficiency, sleep disturbance, use of sleeping medications and daytime dysfunction. To be particular, the subjective sleep quality, sleep latency and daytime dysfunction were the three most severely affected domains in the anxious or depressive patients.

### Risk or protective factors of impaired sleep quality in PN patients

As is shown in Table 1, impaired sleep quality was more prevalent in females than in males (49.4% vs 37.4%, *P* = 0.001). Likewise, older people (aged 50 and above) tended to have an impaired sleep quality than the youngers (51.5% vs 40.2%, *P* = 0.002). Interestingly, patients who had never smoked had a worse sleep quality (*P* = 0.040), although it may be related to the fact that 77.0% of the never-smoker were women. The results also revealed that patients who underwent CT scan recently due to physical discomfort were more likely to report poor sleep quality (*P* = 0.045). We observed that patients with nodule progression (*P* = 0.045) and multiple nodules (*P* = 0.004) exhibited poorer sleep quality after receiving imaging reports or completing their outpatient consultations. Otherwise, the family history of malignancy, subjective symptoms, the size of PN and the initial impression from doctors were found to have no significant influence on the sleep quality (with all *P* > 0.05).

**Table 1:** Analysis for the demographic and clinical factors of pulmonary nodules patients' sleep quality

Variable	Total (*n* = 731)	Good sleep quality group (*n* = 403)	Impaired sleep quality group (*n* = 328)	*P* value
Age				0.002^a^
< 50	428 (58.5%)	256 (59.8%)	172 (40.2%)	
≥ 50	303 (41.5%)	147 (48.5%)	156 (51.5%)	
Gender				0.001^a^
Male	278 (38.0%)	174 (62.6%)	104 (37.4%)	
Female	453 (62.0%)	229 (50.6%)	224 (49.4%)	
Smoking status				0.040^a^
Current	49 (6.7%)	35 (71.4%)	14 (28.6%)	
Ever	116 (15.9%)	67 (57.8%)	49 (42.2%)	
Never	566 (77.4%)	301 (53.2%)	265 (46.8%)	
Family hx of malignancy				0.567^a^
No	328 (44.9%)	177 (54.0%)	151 (46.0%)	
Yes	403 (55.1%)	226 (56.1%)	177 (43.9%)	
Subjective symptoms				0.062^a^
No	479 (65.5%)	276 (57.6%)	203 (42.4%)	
Yes	252 (34.5%)	127 (50.4%)	125 (49.6%)	
Reasons of CT workup				0.044^a^
Health check-up	218 (29.8%)	126 (57.8%)	92 (42.2%)	
Follow-up exam for PN	371 (50.8%)	212 (57.1%)	159 (42.9%)	
Physical discomfort	142 (19.4%)	65 (45.8%)	77 (54.2%)	
Number of pulmonary nodules				0.004^a^
Solitary	344 (47.1%)	209 (60.8%)	135 (39.2%)	
Multiple	387 (52.9%)	194 (50.1%)	193 (49.9%)	
Size of pulmonary nodule				0.566^b^
Mean ± SD	7.99 ± 4.08	7.88 ± 3.93	8.13 ± 4.25	
Time from the date of detection				0.036^a^
0–6 months	410 (56.1%)	219 (53.4%)	191 (46.6%)	
7–12 months	109 (14.9%)	51 (46.8%)	58 (53.2%)	
12–24 months	116 (15.9%)	73 (62.9%)	43 (37.1%)	
> 24 months	96 (13.1%)	60 (62.5%)	36 (37.5%)	
Initial impression of doctors				0.376^a^
Benign	244 (33.4%)	138 (56.6%)	106 (43.4%)	
Indeterminate	272 (37.2%)	141 (51.8%)	131 (48.2%)	
Malignant	215 (29.4%)	124 (57.7%)	91 (42.3%)	
HADS-Anxiety				< 0.001^a^
Not Anxiety (0–7)	430 (58.8%)	278 (64.7%)	152 (35.3%)	
Anxiety (≥ 8)	301 (41.2%)	125 (41.5%)	176 (58.5%)	
HADS-Depression				< 0.001^a^
Not Depression (0–7)	526(72.0%)	326 (62.0%)	200 (38.0%)	
Depression (≥ 8)	205(28.0%)	77 (37.6%)	128 (62.4%)	

*HADS* hospital anxiety and depression scale, *hx* history

^a^chi-square test

^b^Mann–Whitney U test

Through the univariate analysis, variables with *p* < 0.1 were screened out for multivariate analysis. Multivariate binary logistic regression suggested that age ≥ 50 years old (OR 1.88, 95% CI 1.35–2.58; *P* < 0.001), female (OR 1.56, 95% CI 1.05–2.33; *P* = 0.028), detection of nodule for 7–12 months (vs for more than 24 months, OR 2.14, 95% CI 1.18–3.89, *P* = 0.013) anxiety (OR 1.78, 95% CI 1.17–2.71; *P* = 0.007) and depression states (OR 1.84, 95% CI 1.16–2.92; *P* = 0.010) were independent risk factors of impaired sleep quality in PN patients (Table 2).

**Table2:** Multivariate binary logistic regression analysis for independent factors of sleep quality among PN patients

Variables	OR (95%CI)	*P*-value
Age (reference: < 50)	1.88 (1.35, 2.58)	< 0.001
Gender (reference: male)	1.56 (1.05, 2.33)	0.028
Smoking status (reference: never smoke)		
Current	0.57 (0.27, 1.20)	0.139
Ever	1.02 (0.62, 1.68)	0.951
Subjective symptoms (reference: no)	1.14 (0.82, 1.30)	0.441
Reasons of CT workup (reference: physical discomfort)		
Health check-up	0.64 (0.41, 1.03)	0.064
Follow-up exam for PN	0.66 (0.43, 1.02)	0.061
Number of PNs (reference: solitary)	1.31 (0.96, 1.80)	0.089
Time from date of detection(reference: > 24 months)		
0–6 months	1.65 (0.99, 2.73)	0.053
7–12 months	2.14 (1.18, 3.89)	0.013
12–24 months	1.78 (0.72, 2.36)	0.382
HADS-A (reference: not anxiety)	1.78 (1.17, 2.71)	0.007
HADS-D (reference: not depression)	1.84 (1.16, 2.92)	0.010

*HADS* hospital anxiety and depression scale, *hx* history

## Discussion

To the best of our knowledge, this study was the first investigation to explore the sleep conditions among patients with PNs, and hopefully to guide the direction of maintaining mental health and improving sleep quality among the emotionally sensitive population. The present study demonstrated that 44.9% of the PN patients suffered from impaired sleep quality and a much higher prevalence was observed in anxiety or depression patients. Compared with the prevalence of impaired sleep quality in the general population, sleep quality of PN patients was significantly worsen especially in patients with psychological distress [13]. Statistical analysis revealed a robust association between impaired sleep quality and the anxiety or depression states. Sleep disturbance is an ongoing and rapidly evolving public health concern in China and sleep quality of PN patients deserves more attention.

Female patients with PNs tend to report worsen sleep quality than male patients. A similar profile was found in a large meta-analysis among the general population [14]. Bin Zhang et al. [14]. reported a risk ratio of 1.41 for sleep disturbance in women compared with men, which also increased with age. Consistently, age is another independent risk factor for impaired sleep quality in our study. More than a half of patients aging 50 and over had impaired sleep quality, which was similar to that reported by Sho Nakakubo et al. [15]. Interestingly, our previous research has shown that younger people are more anxious than the elders after the detection of PNs [16]. Although anxious patients are more vulnerable for sleep disturbance, it cannot be ignored that normative aging is associated with deficits in sleep physiology [17].

Our study also found that patients with PNs which detected in the short term were more likely to experience impaired sleep quality, while those with PNs detected for more than two years had better sleep quality compared to the former. This finding is consistent with the previous research on dynamic psychological changes in patients with PNs [18, 19]. In the NELSON trial, patients who received an indeterminate result experienced increased lung-cancer-specific distress in the short term [20], but this unfavorable effect was resolved at the 2-year follow-up [19]. This psychological status was similar to the sleep quality of our study at the corresponding time point. However, it is worth noting that patients with nodules detected for 6–12 months reported the worst sleep quality, even compared to those who were detected within half a year. Clinically, patients often undergo a second examination of CT at 6–12 months [21], which may lead to restlessness and concern while waiting for the results to be confirmed, ultimately affecting sleep quality. Despite this, these concerns are often alleviated with prolonged follow-up.

We validated that both anxiety and depression were significantly associated with impaired sleep quality. Substantial studies have also confirmed dual-direction effects between sleep quality and psychological status [18–20]. Sleep disturbance is a common symptom of mental disorders while at the same time high levels of anxiety and depression are usually found in patients with impaired sleep quality [21]. This will develop a vicious circle raising concerns over the escalating burden of PNs. Patients with psychological distress tend to choose more radical and aggressive treatment modalities, which may result in misdiagnosis, removal of benign tumors, as well as the significant waste of medical resources [22, 23]. To improve the psychological status and sleep quality of PN patients, some institutions have offered several good management strategies which are worth learning. At Brooke Army Medical Center, patients with solid lung nodules were provided with a brochure that explained the safety and favorable outcomes to alleviate their anxiety [24]. Daniel C Wiener et al. improved patient compliance and eliminated the fear of cancer through tailoring patient-centered communication [25].

As demonstrated in Fig. 2, anxiety and depression groups showed worsen performance in all dimensions compared with normal group. Among them, daytime dysfunction was affected the most, manifesting as having trouble staying awake and lack of enthusiasm. Such a condition exerts a tremendous impact on personal life and work life, which indicates the necessary of early identification and intervention of psychological problem. In our study, the result shows that impaired sleep quality usually accompanies with and precedes anxiety or depression. Therefore, patients who have not yet reached a diagnosis of anxiety or depression should also receive early intervention if sleep quality is impaired. Additionally, assessment of sleep quality can be used to evaluate potential psychological distress to some extent.

Assessment of sleep quality and psychological states was incorporated into perioperative care of thoracic surgery since poor sleep quality associated with anxiety might increase the risk of postoperative delirium [26]. At present, the management of pulmonary nodule relies mostly on imaging assessment without the emphasis of evaluating the sleep quality and psychological states. The most important concern is that sleep disturbance interacting with psychological distress may promote the malignant development of nodules via weakening of immune response. A series of research have demonstrated subsolid nodules that remained stable for decades fits the theory of equilibrium phase of immunoediting. Thus, functional adaptive anti-tumor immunity but impaired innate anti-tumor immunity is potentially helpful to maintain its dormant behavior [7, 27, 28]. However, chronic sleep disturbance would impair the anti-tumor response and break the balance of the immune status [6, 8, 29]. Therefore, keeping a healthy psychological state and good sleep quality is the most effective treatment modality in some way. For the sake of the best outcome for patients, physicians should take these fundamental points into consideration, not only the imaging results but also the patient’s psychological health.

### Limitations

Several limitations in our study should be acknowledged. Firstly, this study did not collect detailed information about the timing of nodule detection to clearly demonstrate the changes in the quality of sleep among patients with PNs, given its cross-sectional study design. Secondly, anxiety, depression and sleep quality were evaluated using self-report questionnaire, which rendered the results somewhat subjective. Thirdly, all patients were recruited from outpatient clinic, therefore our results may not be generalizable to patients in other clinical settings.

## Conclusions

Impaired sleep quality is prevalent among patients with PNs. Elder people and women are more susceptible to develop sleep disorders. There is a tendency that patients with PNs are more likely to experience poor sleep quality at short-term, whereas this adverse effect seems to be resolved at long-term follow-up. Anxiety and depression, as independent risk factors for sleep disorders, are strongly correlated with sleep quality. Based on the finding of impaired sleep quality and psychological health, screening for psychological and sleep problems in PN patients will be of great clinical benefit.

## Data Availability

All data used and/or analyzed during the current study are available from the corresponding author on reasonable request.

## Abbreviations

PN: Pulmonary nodule
HADS: Hospital Anxiety and Depression Scale
PSQI: Pittsburgh Sleep Quality Index
LDCT: Low-dose computer tomography
PROMs: Patient Reported Outcome Measures
